# 

**Published:** 1894-02-24

**Authors:** 


**Notice to Advertisers and Subscribers.**

All Advertisements, Orders for Copies of THE HOSPITAL, and Business Communications should be addressed to The Publisher, *not to the Editor*, THE HOSPITAL, 428, Strand, W.C.

The Cost of Subscription, post free, is as follows:—Per week, 2½d.; per quarter, 2s. 8d.; per half-year, 5s. 3d.; per annum, 10s. 6d. Foreign:—Per Annum, 12s. 8d.; payable, in all cases, in advance.

**OUR NEW OFFICES.**

428, Strand, W.C., will henceforth be the Office of this Journal.

**Notices of Births, Deaths, and Marriages** are inserted in this column at a charge of 2s. 6d. for an announcement not exceeding four lines.

**NOTICE TO CORRESPONDENTS.**

All MS., Letters, Books for Review, and other matters intended for the Editor should be addressed THE EDITOR, THE LODGE, PORCHESTER SQUARE, LONDON, W.

The Editor cannot undertake to return rejected MS., even when accompanied by stamped directed envelope.

**"Burdett's Hospital Annual," 1893.**

Fourth year of publication. 700 pp. Boards, gilt lettering. A most complete guide to British, Colonial, Indian, and American Hospitals, Dispensaries, Nursing and Convalescent Institutions, and Asylums. Price 5s. Published by THE SCIENTIFIC PRESS (Limited), 428, Strand, W.C.

**NOTICE.—The Index for Vol. XIV. (ending Sept. 30th, 1893) is on sale at the Office of this Journal, price 2½d., post free.**

### BOOKS RECEIVED.

Griffith, Farran and Co.

"Prior Rahere's Rose."

H. K. Lewis, London.

"Sprains, their Consequences and Treatment." By C. W. Mansell Moullin, M.D. Second edition.

"Dwelling Houses, and their Sanitary Construction and Arrangements." By W. Corfield, M.D., M.A.

Messrs. J. and A. Churchill, London.

"Index Pathologicus for the Registration of the Lesions recorded in Pathological Records or Case books of Hospitals and Asylums." By James Howden, M.D.

"The Ophthalmoscope." A Manual for Students. By Gustavus Hartridge, F.R.C.S. Second edition.

Messrs. John Wright, Bristol.

"Notes on Nursing in Eye Diseases. By C. S. Jeaffreson, M.D.

**Periodicals and Pamphlets.**—The Ladies' Gazette of Fashion, the Savannah Medical Journal, the Insurance Observer, Guy's Hospital Gazette.

### Medical Vacancies and Appointments.

### APPOINTMENTS.

S. E. Atkins, L.R.C.S.I., L.S.A.Lond., appointed Medical Officer to the Dalwood, Kilmington, Stockland, and Membury Districts of the Axminster Union. M. R. J. Behrendt, L.R.C.P. Edin., L.R.C.S.Edin., appointed Medical Officer to the Scunthorpe Local Board. F. R. Blaxall, M.D.Lond., D.P.H.Camb., appointed Lecturer on Bacteriology at the Westminster Hospital. G. Braide, L.R.C.P.Lond., M.R.C.S.Eng., appointed Medical Officer to the No. 2 District of the Warrington Local Board. W. C. Haswell, M.B., B.S.Durh., appointed Resident Medical Officer to the Public Dispensary, Leeds. G. A. Hickey, L.R.C.S.I.&L.M., appointed Medical Officer to the New Ross Union. E. W. Hope, M.D., appointed Medical Officer of Health for Liverpool. J. Howell-Griffiths, M.B., B.S.Lond., M.R.C.S., L.R.C.P., appointed Second Assistant Medical Officer to the Greenwich Union Infirmary. C. G. Lee, M.R.C.S.Eng., appointed Honorary Aural Surgeon to the Royal Southern Hospital, Liverpool. A. D. Milne, M.B.Aberd., appointed House Surgeon to the Children's Hospital, East End Branch, Sheffield. J. Niven, M.A.Aberd., M.A.Camb., M.B., appointed Medical Officer of Health for the City of Manchester. F. W. Perry, L.R.C.P.I., L.R.C.S.I., L.M.Dub., appointed House Surgeon to the Pendleton Brunen Dispensary. J. A. F. Sawyer, L.R.C.P.I., L.R.C.S.I., reappointed Medical Officer to the Clevedon Local Board. J. Stobo, L.R.C.P.Edin., L.R.C.S.Edin., appointed Medical Officer to the Southwick Local Board. R. Williams, M.R.C.S., appointed Ophthalmic Surgeon to the Royal Southern Hospital, Liverpool.

### VACANCIES.

### Resident Medical Officers.

Belgrave Hospital for Children, 77 and 79, Gloucester Street, Pimlico, S.W. House Surgeon. Board, lodging, fuel, and light provided. Applications to Percy Gates, Honorary Secretary, by March 7th.

Bury Dispensary Hospital, Bury, Lancashire. Junior House Surgeon. Salary £60 per annum, with board, residence, and attendance. Testimonials to the Secretary, Henry Webb, Brentwood, Bury.

City of London Hospital for Diseases of the Chest, Victoria Park, E. Resident Medical Officer. Salary £100 per annum, with board, &c. Applications to the Secretary, T. Storrar-Smith.

Hospital for Women and Children, Leeds. House Surgeon for less than twelve months. Salary £75 per annum. Applications to the Secretary of the Faculty.

County Asylum, Rainhill, Liverpool. Assistant Medical Officer; unmarried, and not more than 30 years of age. Salary £100 a year, with prospect of an annual rise of £25 up to £200, with further increase according to promotion, together with furnished apartments, board, attendance, and washing. Applications and testimonials to the Medical Superintendent.

County Lunatic Asylum, Sneinton, Nottingham. Assistant Medical Officer; unmarried. Salary £100 per annum, rising £10 annually to £150, board, lodging, washing, and attendance. Applications to the Chairman of the Committee of Visitors by February 27th.

Female Lock Hospital, Harrow Road, W. Assistant House Surgeon. Board and lodging, but no salary. Appointment for twelve months. Applications and testimonials to the Secretary.

French Hospital and Dispensary, 172, Shaftesbury Avenue, W.C. Resident Medical Officer; must speak French. Salary £80 per annum, with board, furnished rooms, and attendance. Applications and testimonials to the Secretary, F. Sord, by March 1st.

London Temperance Hospital, Hampstead Road, N.W. Assistant Resident Medical Officer. Appointment for six months. No salary, but residence in the hospital, board and washing, and an honorarium of five guineas. Applications and testimonials to E. Witson Taylor, Secretary, by March 8th.

Royal South Hants Infirmary, Southampton. House Surgeon. Salary £100 per annum, with board and lodging. Applications with testimonials to the Secretary, T. A. Fisher-Hall, by March 10th.

Royal Surrey County Hospital, Guildford. House Surgeon. Salary £80 per annum, with board, lodging, and laundry. Applications to the Honorary Secretary by March 10th.

Staffordshire County Infirmary, Stafford. Assistant House Surgeon. No salary, but board, lodging, and washing. Applications to House Surgeon.

### Non-Resident Medical Officers.

Bacteriological Institute, Cape Colony. Medical Assistant. Salary £350 per annum. Successful candidate will be provided with a free passage (first class) to the colony. Apply by letter to Mr. Charles Loudon, W.S., 54, Queen Street, Edinburgh.

East London Hospital for Children, Shadwell, E. Pathologist and Registrar. Honorarium £40 per annum. Applications to the Secretary, Thomas Hayes, by February 27th.

County Borough of Oldham. Medical Officer of Health. Salary £400 per annum. Applications, with particulars of qualifications, to be sent to the Town Clerk, A. Nicholson, by February 26th.

Parish of Paddington. Medical Officer of Health. Salary £600 per annum. Applications and testimonials to the Vestry Clerk, Frank Dethridge, Vestry Hall, Harrow Road, W., by February 26th.

